# Symptom and Performance Validity Measures in the Clinical Assessment of Adult ADHD: What Do We Learn from Network Analysis?

**DOI:** 10.1177/10870547251348779

**Published:** 2025-06-27

**Authors:** Anselm B. M. Fuermaier, Oliver Hirsch, Björn Albrecht, Mira-Lynn Chavanon, Hanna Christiansen

**Affiliations:** 1 University of Groningen, The Netherlands; 2 FOM University of Applied Sciences, Siegen, Germany; 3 Philipps University Marburg, Germany

**Keywords:** adult ADHD, network analysis, performance validity, symptom validity, neuropsychological assessment

## Abstract

**Background:**

First-time diagnoses of attention-deficit/hyperactivity disorder (ADHD) in adults can be challenging due to diverse methodologies available for assessment, and the choices clinicians need to make about how to interpret diagnostic criteria. Network analysis is a statistical approach that has received growing attention in clinical research of recent years. It has the potential to aid visualization and illustrate the intricate relationships between the wide range of clinical measures.

**Aim:**

The goal of the present study is to examine the value of network analysis on a sample of *N* = 896 adults newly diagnosed with ADHD in an outpatient referral context.

**Method and Results:**

The network depicts the interrelationship of a comprehensive set of measures and test variables, including symptom self- and other-reports, cognitive tests, motor activity, as well as measures of symptom and performance validity.

**Conclusion:**

Our network analysis supports ADHD symptom clusters with distinct networks of motor activity and attention/impulsivity and reflects the mode of assessment, i.e., neuropsychological versus self- and observer-ratings. The network further depicts the dissociable role of symptom and performance validity measures, and the different nature of embedded and freestanding validity tests. We discuss the future application of network analysis in clinical research on ADHD.

## Introduction

Attention-deficit/hyperactivity disorder (ADHD) is an early-onset neurodevelopmental disorder that persists into adulthood in about 30% to 60% of the affected individuals (American Psychiatric Association, 2013). A sizable number of cases do not present earlier than in early- or mid-adulthood for diagnostic evaluation (Sibley, 2021), which poses a challenge to clinicians if childhood ADHD has not been formally established and requires clinicians to undertake a comprehensive evaluation of current and historical symptoms and impairments (Kooij et al., 2019). Empirically informed guidelines for first-time adult ADHD diagnoses include a range of assessment approaches and instruments in order to promote accurate diagnoses and keep the number of false-positives and negatives as low as possible (Sibley, 2021). Structured clinical interviews following DSM criteria for actual and retrospective early-onset ADHD symptoms commonly form the core of the diagnostic assessment. A diagnostic interview is often accompanied by self- and other report questionnaires to quantify experienced symptoms and impairments (Guo et al., 2021; Harrison & Edwards, 2023). Examples of widely distributed and well-validated instruments are the Conners’ Adult ADHD Rating Scales (Christiansen et al., 2014; Conners et al., 1999) and the Weiss Functional Impairment Rating Scale (WFIRS) (Weiss et al., 2018), which offer empirically validated and normed versions for both examinees themselves and their informants. A marked discrepancy between self- and other reported symptom severity may give indication for a possible lack of insight (of the examinee or the informant) or noncredible symptom report.

Because ADHD is associated predominantly with cognitive difficulties, the clinical evaluation often includes a standardized assessment with neuropsychological performance tests; see (Onandia-Hinchado et al., 2021) for a review on cognitive difficulties in adult ADHD. Even though neuropsychological tests are of no use for the differential clinical diagnosis of adult ADHD (Barkley, 2019; Guo et al., 2023), a thorough assessment of cognitive strengths and weaknesses can be of added value for understanding the examinee’s level of functioning and guide treatment planning and supporting its evaluation (Fuermaier et al., 2019; Mapou, 2019). Tests for sustained attention and vigilance are among the most sensitive measures to reveal cognitive impairments in adults with ADHD, as demonstrated by a large body of empirical research on ADHD in children, adolescents, and (older) adults, see (Fuermaier et al., 2022) for an overview and cross-comparison, and as advocated by an international consensus study (Fuermaier et al., 2019). The cognitive assessment of sustained attention is commonly accomplished by variants of continuous performance tests (CPT), for which a wide variety of instruments have become available for clinicians and researchers (Albrecht et al., 2015). Tests of (sustained) attention also provide implications for higher-order cognitive functions (e.g., executive functions, memory) that build upon attention (Butzbach et al., 2019), however, interestingly, cognitive deficits as assessed in performance tests appear to be only marginally correlated with results of self-reported cognitive deficits in daily life activities and seem to represent distinct clinical features (Fuermaier et al., 2015; Guo et al., 2021).

Besides inattention and impulsivity, motor hyperactivity is defined as one of the core symptom criteria of ADHD (as observed most pronouncedly in children). For this reason, test developers and researchers found ways to provide the integrated assessment of cognitive functions (i.e., inattention and impulsivity in variants of a CPT) with motor behavior (Hirsch & Christiansen, 2017; Ulberstad, 2012) for clinical use.

Accordingly, the Quantified Behavior Test plus (Qb+) is a 20-minute CPT variant measuring sustained attention and impulsivity in a computerized working memory paradigm, combined with a simultaneous infrared motion tracking system of the participant’s head movements. Next to commonly extracted CPT performance measures (i.e., reaction time, variability of reaction time, errors of omissions and commissions) and activity parameters indicating the quantity of movements of the examinee during cognitive task performance, the Qb+ provides age- and sex-specific norms.

Besides psychopathological and neuropsychological assessment, a thorough clinical examination should also consider the degree to which reported problems reflect true experiences of the examinee (with so-called symptom validity tests, SVT) and test performance reflect true cognitive abilities (with so-called performance validity tests, PVT). To this end, clinical practice distinguishes between embedded and stand-alone measures. Embedded SVTs/PVTs reflect measures that are embedded in and derived from existing measures of functioning or test performance (e.g., a clinical rating scale, or cognitive test). Stand-alone (free-standing) SVTs/PVTs, in contrast, are developed for the sole purpose to measure validity of responses or test performance, but without giving information about clinical functioning. A growing and conclusive body of research in the least one to two decades demonstrate non-trivial numbers of noncredible symptom report and test performance in adult ADHD evaluations (Dong et al., 2023; Hirsch & Christiansen, 2018; Ovsiew et al., 2023). Interpretations of current SVT and PVT outcomes are not straightforward, as the indication of cognitive underperformance and/or symptom overreporting do not allow for implications on the underlying reasons. Further, SVTs and PVTs showed only limited correspondence in ADHD and related assessment settings, but seem to represent largely different forms of validity assessment, each carrying unique information (Van Dyke et al., 2013).

Diagnostic challenges of first-time adult ADHD diagnoses and their clinical evaluation result from the various methodologies that are available for the assessment and the choices clinicians need to make about how they interpret diagnostic criteria (Sibley, 2021). The wealth of available assessment approaches, instruments, and their inter-relations may be difficult to oversee for many practitioners. This can be partially explained by past research that only provided snapshots of relationships between a few specific items and constructs. In such studies, statistical analysis techniques typically challenge clinicians and clinical researchers unfamiliar with statistical data, making it difficult for them to obtain a comprehensive picture of ADHD. In this context, Network analysis could make a contribution in visualizing and comprehensively depicting the relationships within and between the discussed assessment approaches and their impact on each other. A network consists of nodes representing all variables of the assessment, and edges connecting these nodes which represent their relationships (Borsboom & Cramer, 2013).

In addition, a node’s Expected Influence shows how strongly it affects other nodes in the network. We used the one-step expected influence algorithm which aims to assess a node’s influence with its immediate neighbor (Bringmann et al., 2019; Guo et al., 2023; Opsahl et al., 2010; Robinaugh et al., 2016).

On these grounds, the goal of the present study is to demonstrate the value of network analysis on a large clinical sample of adults assessed for adult ADHD with a comprehensive battery of symptom self- and observer-reports, cognitive test performance, motor activity, as well as measures of symptom validity, i.e., the Infrequency Index of the CAARS (CII) (Suhr et al., 2011) and performance validity, i.e., the Amsterdam Short Term Memory Test (ASTM) (Schmand & Lindeboom, 2005). For this purpose, a large data set (*N* = 896) of routine clinical data was collected between 2011 and 2019 in an outpatient referral context, see Hirsch and Christiansen (2018) and Hirsch et al. (2022) for previous research on subsets of this data.

We expect distinct networks of nodes for self- and other reported symptomatology, cognitive test performance, and motor behavior. Further, we expect measures of symptom validity to be embedded in the network of self- and other reports and measures of performance validity to be embedded in the network of performance measures. However, because symptom and performance validity do not represent information on clinical (dys) functioning, their Expected Influence is expected to be low compared to measures of functioning and test performance. Within their networks of nodes, the CII, as an embedded SVT, is expected to be associated stronger with self- and other reports than the ASTM, as a stand-alone PVT, is associated with measures of attention and impulsivity.

## Materials and Methods

### Sample

We refer to the two original samples from the studies by Hirsch and Christiansen (2018) (*N* = 196) and Hirsch et al. (2022) (*N* = 700), whereby in the latter study the former sample was also used for classification. As the study by Hirsch et al. (2022) did not show any substantial differences in noncredible symptom report or test performance between the two samples, both were combined to form a total sample, which is described below. Patients with ADHD were individuals newly diagnosed at the adult ADHD outpatient clinic at University of Marburg, Germany. They were all medication-naïve and examined by experienced, licensed clinical psychologists relying on a detailed clinical history, and on several structured diagnostic interviews for ADHD based on DSM-IV and ICD-10. The Conners’ Adult ADHD Rating Scales (CAARS-L self- and observer-ratings), and the Qb+ (Hirsch & Christiansen, 2017; Ulberstad, 2012) were also used to confirm the diagnosis. The Amsterdam Short Term Memory Test (ASTM) was additionally applied as a symptom validity measure. The data was collected between February 2011 and December 2019.

In the current sample, there were 541 men (60.4%) with a mean age of 32.2 years (*SD* = 9.9, range = 18–60 years) and 355 women (39.6%) with a mean age of 34.2 years (*SD* = 11.1, range = 18–60 years). Of these, 469 (52.3%) completed Grammar school, 250 (27.9%) Secondary School, 155 (17.3%) received a Basic Schooling Degree, and 22 (2.5%) had no school degree. All were German residents with German as their first language.

### Ethical Statement

The authors assert that all procedures contributing to this work comply with the ethical standards of the relevant national and institutional committees on human experimentation and with the Helsinki Declaration of 1975, as revised in 2013. The study was approved by the local ethics committee of the University of Marburg, Germany. All participants of both samples gave written informed consent. All participants were provided with a short study description.

### Measures

#### Conners’ Adult ADHD Rating Scales (CAARS-L: S and CAARS-L)

The CAARS-L: S and CAARS-L: O were used in this study. The German version of the CAARS-L: S evaluates symptoms of ADHD in individuals aged 18 years or older, utilizing a Likert-type scale (0 = not at all/never to 3 = very much/very frequently). The extended version comprises 66 items; however, only 42 items were considered in the initial factor analysis conducted by Conners, Erhardt, and Sparrow (Conners et al., 1999) due to statistical constraints imposed by the authors. Their analysis identified four factors: inattention/memory problems, hyperactivity/restlessness, impulsivity/emotional lability, and problems with self-concept. Subsequent confirmatory factor analyses conducted on the German version, involving both healthy adults and those with ADHD, substantiated this factor structure (Christiansen et al., 2011, 2013). Age, gender, and years in education significantly influenced the four subscales, revealing that symptom severity decreased with age, males scored higher on hyperactivity and sensation-seeking behavior, and females scored higher on problems with self-concept. Individuals with less education exhibited higher overall symptom ratings. Test-retest reliability ranged from .85 to .92, with high sensitivity and specificity across all four subscales. The CAARS-L: S is considered a reliable and cross-culturally valid tool for assessing current ADHD symptoms in adults (Christiansen et al., 2012). Similar reliability and validity are observed in the observer version, CAARS-L: O, where a person closely acquainted with the participant rates the same items (Christiansen et al., 2014). The hypothesized factor structure was confirmed, and the observer version exhibited sound statistical criteria. The maximum scores for the subscales are as follows: Inattention/Memory Problems = 36, Hyperactivity/Restlessness = 36, Impulsivity/Emotional Lability = 36, and Problems with Self-Concept = 18. Norms based on gender and age, along with *T*-scores (*M* = 50, *SD* = 10), indicate that scores ≥ 65 represent the 85th percentile and above (Christiansen et al., 2014). We utilized the Inattention/Memory Problems, Hyperactivity/Restlessness, Impulsivity/Emotional Lability, and Problems with Self-Concept subscales from both the self and observer versions. We calculated a validity index known as the Infrequency Index of the CAARS (CII), as introduced by Suhr et al. (2011). The Infrequency Index involves selecting 12 items from the self-version of the Inattention/Memory Problems, Impulsivity/Emotional Lability, and DSM-IV Hyperactive/Impulsive Symptoms scales. Specifically, items are chosen if they are endorsed as occurring “pretty much, often” to “very much, very frequently” by 10% or fewer of the total participant sample. The authors suggested a cut score on this index which is indicative of a noncredible symptom report.

#### Quantified Behavior Test Plus (Qb+)

The Quantified Behavior Test Plus (Qb+) is conducted with participants positioned in front of a computer screen, utilizing specialized equipment comprising an infrared camera, a headband with an attached reflective marker, and a responder button. This test, which gained approval from the Federal Drug Administration in 2014 (Dolgin, 2014), serves as a diagnostic tool for ADHD (Edebol et al., 2012; Lis et al., 2010; Söderström et al., 2014). The Qb+ is a Continuous Performance Test (CPT) designed to measure sustained attention through a 1-back working memory task involving the recall of the same object in shape and color. This is coupled with a simultaneous high-resolution motion tracking system, enabling the separate assessment of hyperactivity, inattention, and impulsivity with nine parameters within a 20-minute timeframe. Stimuli presented during the test include a blue circle, a blue square, a red circle, and a red square. Participants are required to press a response key when two identical stimuli are displayed consecutively, necessitating the retention of stimulus information in working memory until the next presentation allows for a matching process (Lis et al., 2010). The target to non-target stimuli ratio is maintained at 25:75.

During the execution of the CPT, the movements of the participant are recorded by an infrared camera tracking a reflective marker affixed to a headband worn by the participant. The camera is positioned approximately 1 m away from the participant, who is seated in front of the computer screen. Participants use a chair with back support but without armrests to ensure a comfortable yet non-reclining posture during testing. The participant’s activities are tracked by reading the coordinates (X and Y) of the headband marker, sampled 50 times per second, with a spatial resolution of 1/27 mm per camera unit.

The Qb+ provides a comprehensive report of nine parameters, categorized into activity and CPT measures. Activity measures include (a) Time Active, representing the percentage of time the subject has moved more than 1 cm/s; (b) Distance, indicating the total distance covered by the reflective headband marker; (c) Area, denoting the surface covered by the headband reflector during the test; (d) Total Number of Micro events, signifying small movements of the reflective marker; and (e) Motion Simplicity, a measure of the complexity of the motion pattern, reported as a percentage. CPT measures comprise (f) Reaction Time (RT), reflecting the average time of all correct responses; (g) RT Variation (RTVar), calculated using the standard deviation of the mean of correct response times, representing the participant’s inconsistency in response times; (h) Omission Errors, indicating the total number of missed targets; and (i) Commission Errors, representing the total number of false hits.

Normative data for both versions of the test (QbTest 6–12 and Qb+) have been established from a sample of 1,307 individuals aged 6 to 60 years, ensuring an even age and gender distribution (Ulberstad, 2012). In the current study, Q scores for the listed parameters were utilized. Q scores for hyperactivity, inattention, and impulsivity reflect the deviation of the participant’s performance (in standardized units) from the mean score of the age-and-gender normed non-ADHD nonclinical group, interpreted similarly to Z scores with a mean of 0 and a standard deviation of 1, have been derived for hyperactivity, inattention, and impulsivity. Sensitivity and specificity are within the acceptable range (Bellato et al., 2024).

#### Amsterdam Short Term Memory Test (ASTM)

The Amsterdam Short Term Memory Test (ASTM) functions as a performance validity test (PVT) designed to evaluate negative response bias and insufficient motivation during psychological assessments (Schmand & Lindeboom, 2005). Presented as an assessment of short-term memory and attention, the ASTM involves displaying five semantically related words for 8 s. Participants are instructed to read the words aloud and memorize them. Subsequently, a simple arithmetic problem is presented. Following this, five words are shown again, including three from the initial set. Participants are tasked with identifying the three previously presented words. A maximum of 90 points can be achieved across thirty tasks. The test exhibits satisfactory reliability, with internal consistency measuring around .90 in various samples. In a sample of mixed neurological patients, the test–retest correlation was .85 within a 1 to 3-day interval.

The ASTM employs a cutoff value to indicate a failed performance. Sensitivity to feigned cognitive dysfunction was reported as 91% in experimental simulants, and specificity to genuine cognitive dysfunction was found to be 89% in neurological patients. Healthy controls aged 9 years and older typically perform exceptionally well on this test. Patients with neurological disorders such as concussion, brain tumors, multiple sclerosis, or difficult-to-treat epilepsy generally navigate the test successfully, assuming they do not possess severe cognitive deficits.

### Statistical Analyses

#### Network Estimation

The network of neuropsychological functions was estimated using the R packages *bootnet* and *qgraph* (Epskamp et al., 2012, 2018). Within these networks, 19 variables were presented as nodes, while the edges represented partial correlation coefficients between neuropsychological functions. These coefficients signify the correlation between two variables after accounting for all other variables in the network (Borsboom & Cramer, 2013). To enhance interpretability and prevent spurious connections, the graphical lasso algorithm, a variant of the widely used regularization algorithm known as the least absolute shrinkage and selection operator (LASSO), was employed for network estimation (Epskamp & Fried, 2018; Tibshirani, 1996). The graphical lasso algorithm regulates the degree of regularization through a tuning parameter (λ), which is determined using the Extended Bayesian Information Criterion (EBIC) (Chen & Chen, 2008; Friedman et al., 2008). The visualization of these networks relied on the Fruchterman–Reingold algorithm (Fruchterman & Reingold, 1991). In the graph generated by the Fruchterman–Reingold algorithm, nodes with stronger connections are positioned closer to each other, and edges between nodes with higher absolute coefficients are depicted with thicker and more saturated colored lines. Due to the non-normal distribution of our data, Spearman correlations were utilized as input (Isvoranu & Epskamp, 2023).

#### Node Centrality Estimation

We assessed the relative importance of variables in the network using node expected influence, a node centrality index that aims to assess a node’s influence with its immediate neighbors based on the one-step expected influence algorithm (Robinaugh et al., 2016). In comparison to node strength, the previously predominant node centrality index representing the sum of the absolute value of connections for a node, node expected influence takes into account both positive and negative connections (Opsahl et al., 2010; Robinaugh et al., 2016). To compute and visualize the expected influence, we employed the centrality, centralityTable, and centralityPlot functions from the qgraph package (Epskamp et al., 2012).

#### Accuracy and Stability Estimation

We evaluated the precision of edge weights and the consistency of the node centrality order. To assess edge weight accuracy, we employed bootstrapping to calculate the 95% confidence intervals (CIs) of the edge weights. Smaller CIs were indicative of a higher accuracy of the order of most edges within the network. For the estimation of node centrality stability, we utilized the correlation stability coefficient (CS coefficient). Following the simulation design by Epskamp et al. [48], CS coefficients exceeding 0.25 denoted moderate stability, while those surpassing 0.5 indicated strong stability. The analyses were conducted using the R package bootnet.

CAARS and ASTM variables were z-transformed using a robust z score transformation based on the median absolute deviation (Iglewicz & Hoaglin, 1993), and Q based norm scores of the Qb+ test were entered in the analyses

All analyses were performed with R version 4.2.3 (R Core Team, 2023) and Rstudio Version 2023.03.0.386 (Poist Team, 2023) by using R packages bootnet version 1.5.6 and qgraph version 1.9.8.

#### Explorative Subgroup Analysis

In explorative analysis, we repeated network analyses on two subsamples, that is, those individuals who (1) passed the ASTM as a PVT, and (2) passed the CII as a SVT. We interpret both networks of nodes in regard to their added value to the understanding of symptom and performance validity measures in comprehensive neuropsychological evaluations of adults with ADHD.

## Results

### Network Estimation

**Figure 1.**
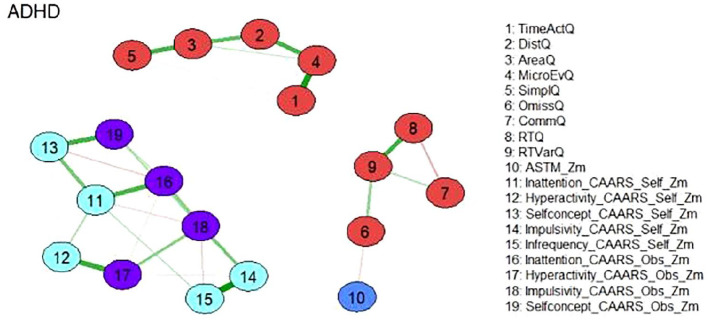
Network of neuropsychological functions for the total ADHD sample (*N* = 896). Neuropsychological test variables are denoted by nodes. Variables originating from the same neuropsychological tests are depicted in identical colors. The connections between nodes are represented by edges, illustrating regularized partial Spearman correlations. Thicker and more saturated colored edges correspond to higher absolute correlations. Positive correlations are denoted by green edges, while negative correlations are indicated by red edges. *Note*. ADHD = attention-deficit/hyperactivity disorder; Q = Q score; self = self-version; obs = observer version; Zm = robust z score transformation based on the median absolute deviation.

Figure 1 shows the estimated network which consists of three separate subnetworks. Two of the subnetworks consist almost exclusively of nodes representing Qb+ variables and reproduce nearly perfectly the postulated factorial structure of the instrument [19]. The first factor “Hyperactivity” is represented by nodes 1 to 5 which are defined by the corresponding variables of this construct (see Figure 1). Commission errors (node 7) belonging to the factor “Impulsivity” is a member of the network consisting of nodes representing the “Inattention” construct of the Qb+ (nodes 6, 8, 9). This seems reasonable, as the confirmatory factor analysis of the Qb+ also revealed a rather weak singlet factor “Inattention”. An external position in this network is occupied by the node representing the ASTM (node 10), which correlates moderately negatively with omission errors after controlling for the other nodes (node 6; see partial Spearman correlations in the Supplemental Material). The third subnetwork exclusively consists of nodes representing CAARS self and observer scales. The corresponding constructs of the self and observer version are located in the immediate vicinity. This indicates the validity of the constructs, as the data results from different sources. An external position in this subnetwork is occupied by the node representing the CAARS Infrequency Index self-version (CII, node 15), which correlates highly positively with the node representing the CAARS Impulsivity self-version (node 14) and moderately with the node representing CAARS Inattention self-version (node 11) after controlling for the other nodes (see partial Spearman correlations in the Supplemental Material).

### Node Centrality Estimation

**Figure 2.**
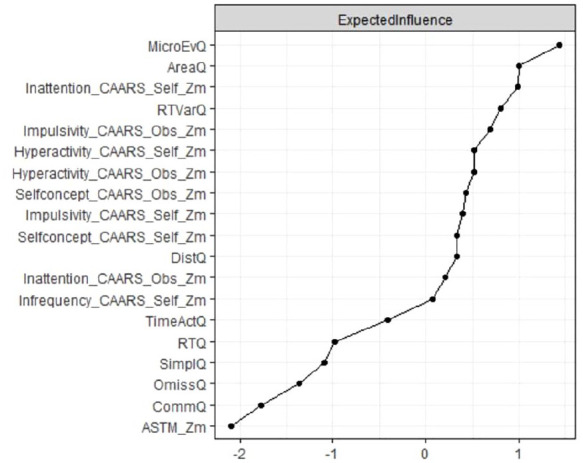
Node expected influence (total ADHD sample; *N* = 896). Elevated standardized z-scores depicted on the x-axis signify increased expected influence, indicating that nodes with higher expected impact exhibit more proximate and robust connections with other neuropsychological test variables in the network.

Node centrality estimations are presented in Figure 2. Nodes with high expected influence are mainly Qb+-related variables of motor hyperactivity and inattention which is also reflected in the high expected influence of nodes representing CAARS subscales covering inattention, impulsivity, and hyperactivity, both as a self-assessment and as an assessment by a significant other.

### Stability Estimation

The assessment of edge weight accuracy disclosed relatively compact and moderate confidence intervals, affirming the accurate estimation of the orders of edge weights (refer to Figure S3 in the Supplemental Material). In terms of node centrality estimation, the CS coefficient was determined to be 0.75 for expected influence, signifying that with 95% certainty, up to 75% of cases could be omitted while still maintaining a correlation with the original centrality exceeding 0.7.

### Explorative Subgroup Analysis

**Figure 3.**
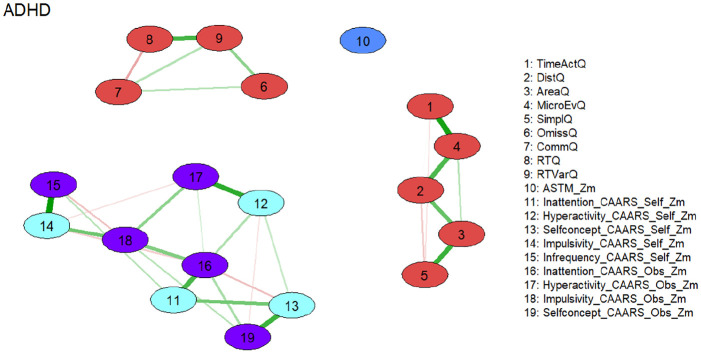
Network of neuropsychological functions for the subgroup of individuals with ADHD passing the performance validity test (ASTM; *N* = 490). Neuropsychological test variables are denoted by nodes. Variables originating from the same neuropsychological tests are depicted in identical colors. The connections between nodes are represented by edges, illustrating regularized partial Spearman correlations. Thicker and more saturated colored edges correspond to higher absolute correlations. Positive correlations are denoted by green edges, while negative correlations are indicated by red edges. *Note*. ADHD = attention-deficit/hyperactivity disorder; ASTM = Amsterdam Short Term Memory Test; Q = Q Score; self = self-version; obs = observer version; Zm = robust *z* score transformation based on the median absolute deviation.

**Figure 4.**
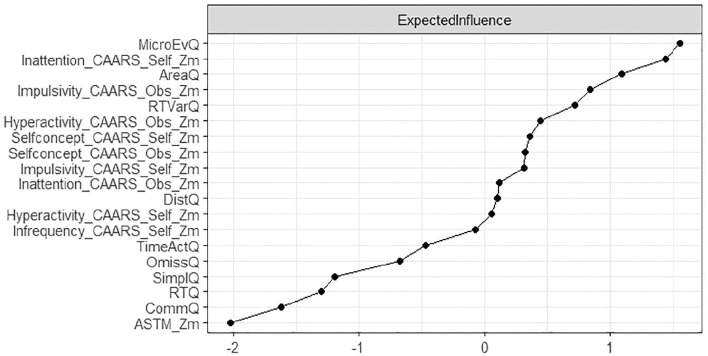
Node expected influence (subgroup passing ASTM; *N* = 490). Elevated standardized z-scores depicted on the x-axis signify increased expected influence, indicating that nodes with higher expected impact exhibit more proximate and robust connections with other neuropsychological test variables in the network.

**Figure 5.**
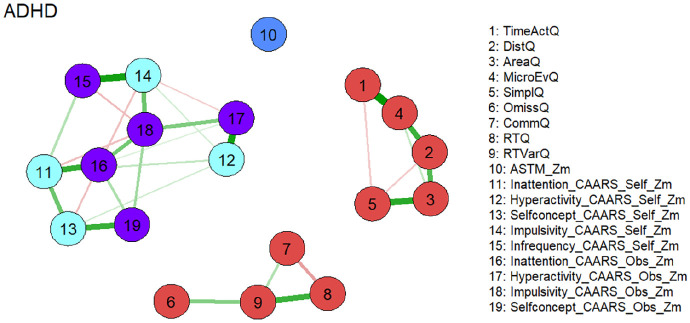
Network of neuropsychological functions for the subgroup of individuals with ADHD passing the symptom validity test (CII; *N* = 570). Neuropsychological test variables are denoted by nodes. Variables originating from the same neuropsychological tests are depicted in identical colors. The connections between nodes are represented by edges, illustrating regularized partial Spearman correlations. Thicker and more saturated colored edges correspond to higher absolute correlations. Positive correlations are denoted by green edges, while negative correlations are indicated by red edges. Abbreviations: ADHD = attention-deficit/hyperactivity disorder; CII = CAARS Infrequency Index; Q = Q Score; self = self-version; obs = observer version; Zm = robust *z* score transformation based on the median absolute deviation.

**Figure 6.**
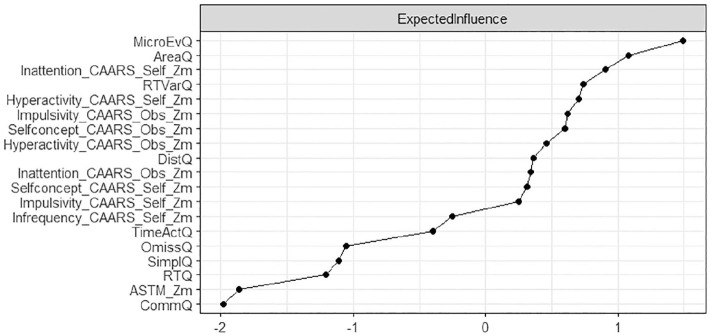
Node expected influence (subgroup passing CII; *N* = 570). Elevated standardized *z*-scores depicted on the x-axis signify increased expected influence, indicating that nodes with higher expected impact exhibit more proximate and robust connections with other neuropsychological test variables in the network.

In explorative analyses, we repeated the network analysis on a sample who passed the ASTM (*N* = 490), as well as a sample who passed the CII (*N* = 570). Network analyses on subgroups revealed a similar overall picture compared to the total group, with three distinct subnetworks representing motor behavior, cognitive test performance, and symptom reports. Results also resembled the total sample with regard to the variables of strongest expected influence. The variability of reaction time was strongest in the subnetwork of cognitive test performance, and self-reported inattention was strongest in the subnetwork of symptom reports. The CII as a SVT was strongly connected in the subnetwork of symptom reports, however, in both explorative subgroup analyses, the ASTM as a PVT lost its connection to the subnetwork of test performance scores (Figures 3–6).

## Discussion

Overall, our results are largely in line with our assumptions. We expected distinct networks of nodes for self- and other reported symptomatology, cognitive test performance, and motor behavior. This is supported by the performed network analyses, as we found three highly stable and distinct networks, two of which encompass neuropsychological assessments, and one with symptoms assessed with the CAARS self- and observer-rating scales. The three networks identified relate to the core symptom of 1) hyperactivity with the variables of motor activity of the Qb+ test; 2) inattention/impulsivity with the corresponding Qb+ test variables; 3) all three core symptoms as rated with the CAARS self- and observer-scales.

Within the network motor activity the variable micro-events, i.e. small movements of the reflective head marker that occur when a position change since the last micro event is greater than 1 mm, has the largest expected influence, indicating that it has the strongest connections to the rest of the network. Those micro events are hard to observe outside the neuropsychological lab and might suggest that subtle hyperactivity may be an important, but probably often neglected characteristic of ADHD, as happens especially for female patients (Beheshti et al., 2021; Bruchmüller et al., 2012; Fraticelli et al., 2022).

The attention/impulsivity network is driven by reaction time variability, i.e., the standard deviation of the mean of correct response times. Reaction time variability in a variety of cognitive tasks is one of the most stable and replicated behavioral correlates of ADHD (Salum et al., 2019), and as a measure of the participant’s performance inconsistency an indicator of (periodic) lapses of attention (Levy et al., 2018; Münger et al., 2021).

As stated above, the network structure of the two Qb+ variables networks fully confirms the established factorial structure of the Qb+ test, which clusters the domains of motor activity and inattention/impulsivity (Hirsch & Christiansen, 2017). While usually hyperactivity and impulsivity are clustered together, i.e., as in classification systems such as the DSM-IV and -5, ICD-11 (World Health Organization, 2025), the current results point to the importance of impulsivity within the domain of inattention, as is observed, for example in symptoms such as mind wandering, jumping from one topic to the next, or difficulties in maintaining a discussion/social interaction (Lanier et al., 2021). This may be a consequence of the 1-back CPT demand (requiring a response to stimulus repetition) and 25 % target probability in the Qb+: if the foregoing or the actual stimulus is missed (inattention) and the actual response may be regarded as random, the likelihood for a commission error equals 75% times the prepotency to respond.

Within the CAARS network, the four main content scales inattention, hyperactivity, impulsivity, and self-concept are included and the self- and observer scales of those subscales show strong corresponding connections. Self-reported inattention showed the strongest influence to other variables of this network. Thus, results also reflect the measurement methods used, as neuropsychological and questionnaire assessments are clustered (Hirsch & Christiansen, 2017). We further expected measures of symptom validity to be embedded in the network of self- and other reports, and measures of performance validity to be embedded in the network of performance measures. This assumption is confirmed, as the CAARS infrequency (CII) index is embedded in the CAARS network via self-reported impulsivity, whilst the ASTM is rather loosely connected to Qb+ omission errors—both with low Expected Influence on the rest of the domains in the network. One may speculate whether SVT and PVT measures, and in particular the ASTM score have limited variance in the assessed sample of confirmed ADHD cases, and accordingly low influence of other parts of the networks, leading to weak connections that may be driven in case of CAARS SVT by item communalities and in case of PVT by common neuropsychological functioning tapped. This also conforms to our expectations that the ASTM, as a stand-alone PVT, has the lowest Expected Influence of all measures, given it is the only measure that does not carry information on (clinical) functioning, but validity of test performance. The CII, as an embedded SVT, has the lowest Expected Influence within the CAARS network. However, the CII, in its network, has more influence compared to the ASTM, which appears plausible for embedded validity measures that are derived from items of clinical instruments (i.e., original CAARS items).

Networks from the exploratory subgroup analyses demonstrated the robustness of the overall model and largely confirmed the findings from the total sample. We observed similar network structures, including variables with strong expected influence, regardless of whether individuals with elevated symptom reporting (CII) or low test performance (ASTM) were excluded. Notably, the CII remained well-connected to other symptom report variables, even when its range was restricted by removing those scoring above the cutoff. This finding supports the notion that the CII, as an embedded SVT, measures a clinically relevant construct, also in individuals whose symptom scores fall within the normal range. In contrast, the ASTM, as a freestanding PVT, does not appear to assess clinically meaningful aspects of functioning. This is reflected by the absence of connections between the ASTM and performance variables once participants with suspiciously low ASTM scores were excluded. We interpret the (weak) connections between the ASTM and test performance observed in the total sample as indicative of noncredible examinees performing below their ability on both the ASTM and the Qb+ (see discussion of continuous performance tests, such as the Qb+, as embedded validity measures; e.g., Ord et al., 2021; Ovsiew et al., 2023). However, when noncredible performance data are removed, this connection disappears. An alternative explanation could be that noncredible performance is sometimes confused with genuine cognitive impairment. Following this reasoning, individuals with severe cognitive deficits and high ADHD symptom severity may fall below the ASTM cutoff despite exerting full effort during the assessment.

### Limitations

Several limitations should be considered when interpreting the findings of this study. The comparability between the earlier and current samples may be constrained by nonspecific differences in recruitment or selection processes, sample characteristics, and the utilized assessment battery. These factors introduce complexities in the interpretation of the study’s results. Owing to variations in the structured clinical interview (WRI vs DIVA) within the assessment battery and differences in neuropsychological tasks (Attention Performance Test Battery, TAP + QbTest vs QbTest alone) between the earlier and current samples, the analyses were limited to utilizing only the measures available in both datasets. Consequently, this resulted in the exclusion of scores from the attention performance test battery (TAP), which was administered solely in the earlier sample (Hirsch & Christiansen, 2018). Notably, significant differences were observed between individuals who passed and failed the performance validity test assessment on variables related to divided attention, sustained attention, and inhibitory control within the TAP test battery.

It is important to note that the present statistical methodology does not allow for the calculation of effect sizes in network analyses. Additionally, it is crucial to emphasize that networks do not imply causal relationships and that the validity of centrality measures in psychological networks is discussed controversially (Bringmann et al., 2019; Dablander & Hinne, 2019).

The current sample is predominantly male with over 60 %, rather young (mean age of 32 years), and well-educated (over 50 % with at least grammar school). Future analyses on gender, age, and educational differences could shed light on which symptoms drive ADHD diagnoses in those different groups and whether there are differences between the networks of males and females, younger and older patients with higher or lower educational attainment.

Finally, we will extend our assessment battery by more symptom and performance validity measures that tap different constructs and domains, so that future analyses will show whether the inclusion of different SVTs and PVTs will result in networks of their own.

## Conclusions

In this paper, we conclude that network analysis appears to be an elegant statistical approach to depict a visual presentation of complex interrelationships of large sets of variables. We argue that the intuitive and accessible interpretation of network analysis advances our understanding of complex multivariate relationships in the neuropsychological assessment of adult ADHD. The graphical presentation of the network of nodes shows that self-reports of cognitive functioning and performance measures of cognition represent distinct and nonredundant sources of information and should not be treated interchangeably. Further, we learn from the position and strength of certain nodes (“Expected Influence”) that reaction time variability in performance tests and self-reported inattention symptoms in symptom reports stand out, advising clinicians to prioritize these variables in their evaluations, and potentially allowing for shorter test batteries. Moreover, we conclude that SVTs and PVTs are poorly related constructs and should be interpreted independently. Network analyses further underlines the dissociation between embedded and free-standing validity measures, leading to distinct applications and limitations. Whereas free-standing validity measures are only loosely connected with clinical measures and represent an own class of instrument, embedded validity tests are derived from clinical measures resulting in strong connections with these instruments. This suggests lower face validity of embedded measures but also a higher risk of misinterpreting genuine pathology as invalid data.

### Future Directions

In future research it would be of interest to extend the currently presented network with aspects of functional impairments, in order to learn about the multivariate interrelationship between symptom clusters and impairments in daily life activities (see Fuermaier et al., 2024, for a discussion on the assessment of impairment in adults with ADHD). Network analysis may further be a suitable approach to compare subgroups of individuals with ADHD that differ in clinical, demographic, or descriptive characteristics, in order to shed light on the heterogeneity of the condition. Finally, it appears worthwhile to explore the stability of the derived network structure in longitudinal research, i.e., after successful treatment or in different developmental stages of the individuals. For such analysis, interpretation of the findings and their clinical implications would benefit from the calculation of effect sizes of network analyses (which is not possible based on current statistical methodology, see Guo et al., 2023), for example to compare global connectivity or expected influence between networks of subgroups.

## Supplemental Material

sj-docx-1-jad-10.1177_10870547251348779 – Supplemental material for Symptom and Performance Validity Measures in the Clinical Assessment of Adult ADHD: What Do We Learn from Network Analysis? Supplemental material, sj-docx-1-jad-10.1177_10870547251348779 for Symptom and Performance Validity Measures in the Clinical Assessment of Adult ADHD: What Do We Learn from Network Analysis? by Anselm B. M. Fuermaier, Oliver Hirsch, Björn Albrecht, Mira-Lynn Chavanon and Hanna Christiansen in Journal of Attention Disorders
