# Supplementary material for: Symptom and Performance Validity Measures in the Clinical Assessment of Adult ADHD: What Do We Learn from Network Analysis?
Source: J Atten Disord. 2025 Jun 27;29(13):1177–89. doi: 10.1177/10870547251348779 (PMC12480614; doi:10.1177/10870547251348779)
Supplement: sj-docx-1-jad-10.1177_10870547251348779 – Supplemental material for Symptom and Performance Validity Measures in the Clinical Assessment of Adult ADHD: What Do We Learn from Network Analysis? [file sj-docx-1-jad-10.1177_10870547251348779.docx]

**Supplemental Material**

**Partial Spearman correlations**

TimeActQ DistQ AreaQ MicroEvQ SimplQ

TimeActQ 1.00000000 0.94666396 0.89777209 0.97784956 0.46732556

DistQ 0.94666396 1.00000000 0.95629512 0.98029206 0.53474037

AreaQ 0.89777209 0.95629512 1.00000000 0.94273920 0.67176878

MicroEvQ 0.97784956 0.98029206 0.94273920 1.00000000 0.51459737

SimplQ 0.46732556 0.53474037 0.67176878 0.51459737 1.00000000

OmissQ 0.28727708 0.30275589 0.32408538 0.29627306 0.29312513

CommQ 0.17952952 0.18654012 0.20225553 0.17551596 0.15960815

RTQ 0.18651989 0.16466312 0.17086743 0.17607083 0.14132189

RTVarQ 0.32518803 0.31864981 0.33558330 0.32858294 0.25412353

ASTM_Zm -0.12815357 -0.13936162 -0.14183266 -0.13109988 -0.10530211

Inattention_CAARS_Self_Zm 0.04779777 0.06385379 0.06452194 0.04143638 0.08595650

Hyperactivity_CAARS_Self_Zm 0.11223448 0.13082633 0.11969613 0.10666944 0.06942114

Selfconcept_CAARS_Self_Zm -0.03381749 -0.03050751 -0.02457119 -0.04348487 0.01855325

Impulsivity_CAARS_Self_Zm 0.15458086 0.15411062 0.16776446 0.15053039 0.06651079

Infrequency_CAARS_Self_Zm 0.18530065 0.18792127 0.19416681 0.18922476 0.08107670

Inattention_CAARS_Obs_Zm 0.04945705 0.07695738 0.07276131 0.05142539 0.09916444

Hyperactivity_CAARS_Obs_Zm 0.13418644 0.14902330 0.13513347 0.12799046 0.07198184

Impulsivity_CAARS_Obs_Zm 0.03974515 0.06011868 0.05538502 0.03846247 0.04422754

Selfconcept_CAARS_Obs_Zm -0.03776934 -0.02615064 -0.02542482 -0.04118680 0.02424011

OmissQ CommQ RTQ RTVarQ ASTM_Zm

TimeActQ 0.287277077 0.17952952 0.186519886 0.325188031 -0.12815357

DistQ 0.302755888 0.18654012 0.164663121 0.318649810 -0.13936162

AreaQ 0.324085380 0.20225553 0.170867425 0.335583302 -0.14183266

MicroEvQ 0.296273056 0.17551596 0.176070833 0.328582943 -0.13109988

SimplQ 0.293125131 0.15960815 0.141321888 0.254123531 -0.10530211

OmissQ 1.000000000 0.25481604 0.310023728 0.516224625 -0.27428331

CommQ 0.254816042 1.00000000 -0.049724375 0.254026721 -0.21490737

RTQ 0.310023728 -0.04972438 1.000000000 0.605221196 -0.11152558

RTVarQ 0.516224625 0.25402672 0.605221196 1.000000000 -0.23716025

ASTM_Zm -0.274283315 -0.21490737 -0.111525578 -0.237160247 1.00000000

Inattention_CAARS_Self_Zm 0.074217296 0.05460248 0.074834822 0.099462737 -0.14785374

Hyperactivity_CAARS_Self_Zm 0.033719500 0.09283094 0.007226957 0.077501674 -0.10431699

Selfconcept_CAARS_Self_Zm 0.011726573 0.04842214 0.015626740 0.036989735 -0.13000913

Impulsivity_CAARS_Self_Zm 0.170023684 0.14949392 0.072394030 0.184334380 -0.13497141

Infrequency_CAARS_Self_Zm 0.188436403 0.11541464 0.100708957 0.204259755 -0.11697820

Inattention_CAARS_Obs_Zm 0.020981650 0.04958167 0.023904952 0.037813343 -0.11342084

Hyperactivity_CAARS_Obs_Zm 0.021796963 0.07547839 -0.019257018 0.085693935 -0.14110815

Impulsivity_CAARS_Obs_Zm -0.001540974 0.10181679 -0.027958101 0.041701719 -0.13808033

Selfconcept_CAARS_Obs_Zm -0.014215945 0.01744304 -0.013110031 0.007259536 -0.09934716

Inattention_CAARS_Self_Zm Hyperactivity_CAARS_Self_Zm

TimeActQ 0.04779777 0.112234480

DistQ 0.06385379 0.130826325

AreaQ 0.06452194 0.119696128

MicroEvQ 0.04143638 0.106669436

SimplQ 0.08595650 0.069421143

OmissQ 0.07421730 0.033719500

CommQ 0.05460248 0.092830937

RTQ 0.07483482 0.007226957

RTVarQ 0.09946274 0.077501674

ASTM_Zm -0.14785374 -0.104316995

Inattention_CAARS_Self_Zm 1.00000000 0.580214139

Hyperactivity_CAARS_Self_Zm 0.58021414 1.000000000

Selfconcept_CAARS_Self_Zm 0.67301692 0.485798140

Impulsivity_CAARS_Self_Zm 0.27134343 0.366238689

Infrequency_CAARS_Self_Zm 0.39413776 0.378992805

Inattention_CAARS_Obs_Zm 0.69898851 0.462351703

Hyperactivity_CAARS_Obs_Zm 0.46414591 0.741762982

Impulsivity_CAARS_Obs_Zm 0.44310994 0.542598335

Selfconcept_CAARS_Obs_Zm 0.53024266 0.393625654

Selfconcept_CAARS_Self_Zm Impulsivity_CAARS_Self_Zm

TimeActQ -0.03381749 0.15458086

DistQ -0.03050751 0.15411062

AreaQ -0.02457119 0.16776446

MicroEvQ -0.04348487 0.15053039

SimplQ 0.01855325 0.06651079

OmissQ 0.01172657 0.17002368

CommQ 0.04842214 0.14949392

RTQ 0.01562674 0.07239403

RTVarQ 0.03698974 0.18433438

ASTM_Zm -0.13000913 -0.13497141

Inattention_CAARS_Self_Zm 0.67301692 0.27134343

Hyperactivity_CAARS_Self_Zm 0.48579814 0.36623869

Selfconcept_CAARS_Self_Zm 1.00000000 0.23696196

Impulsivity_CAARS_Self_Zm 0.23696196 1.00000000

Infrequency_CAARS_Self_Zm 0.28760271 0.79980739

Inattention_CAARS_Obs_Zm 0.46458726 0.13863798

Hyperactivity_CAARS_Obs_Zm 0.37812757 0.23270637

Impulsivity_CAARS_Obs_Zm 0.44583665 0.42433955

Selfconcept_CAARS_Obs_Zm 0.71165986 0.13101069

Infrequency_CAARS_Self_Zm Inattention_CAARS_Obs_Zm

TimeActQ 0.1853007 0.04945705

DistQ 0.1879213 0.07695738

AreaQ 0.1941668 0.07276131

MicroEvQ 0.1892248 0.05142539

SimplQ 0.0810767 0.09916444

OmissQ 0.1884364 0.02098165

CommQ 0.1154146 0.04958167

RTQ 0.1007090 0.02390495

RTVarQ 0.2042598 0.03781334

ASTM_Zm -0.1169782 -0.11342084

Inattention_CAARS_Self_Zm 0.3941378 0.69898851

Hyperactivity_CAARS_Self_Zm 0.3789928 0.46235170

Selfconcept_CAARS_Self_Zm 0.2876027 0.46458726

Impulsivity_CAARS_Self_Zm 0.7998074 0.13863798

Infrequency_CAARS_Self_Zm 1.0000000 0.16564508

Inattention_CAARS_Obs_Zm 0.1656451 1.00000000

Hyperactivity_CAARS_Obs_Zm 0.2200925 0.55740698

Impulsivity_CAARS_Obs_Zm 0.2602214 0.63010216

Selfconcept_CAARS_Obs_Zm 0.1052984 0.59447594

Hyperactivity_CAARS_Obs_Zm Impulsivity_CAARS_Obs_Zm

TimeActQ 0.13418644 0.039745148

DistQ 0.14902330 0.060118680

AreaQ 0.13513347 0.055385022

MicroEvQ 0.12799046 0.038462469

SimplQ 0.07198184 0.044227535

OmissQ 0.02179696 -0.001540974

CommQ 0.07547839 0.101816789

RTQ -0.01925702 -0.027958101

RTVarQ 0.08569393 0.041701719

ASTM_Zm -0.14110815 -0.138080333

Inattention_CAARS_Self_Zm 0.46414591 0.443109943

Hyperactivity_CAARS_Self_Zm 0.74176298 0.542598335

Selfconcept_CAARS_Self_Zm 0.37812757 0.445836652

Impulsivity_CAARS_Self_Zm 0.23270637 0.424339546

Infrequency_CAARS_Self_Zm 0.22009245 0.260221443

Inattention_CAARS_Obs_Zm 0.55740698 0.630102157

Hyperactivity_CAARS_Obs_Zm 1.00000000 0.654071264

Impulsivity_CAARS_Obs_Zm 0.65407126 1.000000000

Selfconcept_CAARS_Obs_Zm 0.44104914 0.575477491

Selfconcept_CAARS_Obs_Zm

TimeActQ -0.037769342

DistQ -0.026150638

AreaQ -0.025424825

MicroEvQ -0.041186802

SimplQ 0.024240112

OmissQ -0.014215945

CommQ 0.017443039

RTQ -0.013110031

RTVarQ 0.007259536

ASTM_Zm -0.099347159

Inattention_CAARS_Self_Zm 0.530242662

Hyperactivity_CAARS_Self_Zm 0.393625654

Selfconcept_CAARS_Self_Zm 0.711659858

Impulsivity_CAARS_Self_Zm 0.131010686

Infrequency_CAARS_Self_Zm 0.105298403

Inattention_CAARS_Obs_Zm 0.594475937

Hyperactivity_CAARS_Obs_Zm 0.441049140

Impulsivity_CAARS_Obs_Zm 0.575477491

Selfconcept_CAARS_Obs_Zm 1.000000000

**centralityTable**

graph type node measure value

1 graph 1 NA TimeActQ Betweenness -0.67504241

2 graph 1 NA DistQ Betweenness 2.12841240

3 graph 1 NA AreaQ Betweenness 0.16599403

4 graph 1 NA MicroEvQ Betweenness 0.16599403

5 graph 1 NA SimplQ Betweenness -0.67504241

6 graph 1 NA OmissQ Betweenness -0.46478330

7 graph 1 NA CommQ Betweenness -0.67504241

8 graph 1 NA RTQ Betweenness -0.67504241

9 graph 1 NA RTVarQ Betweenness -0.39469693

10 graph 1 NA ASTM_Zm Betweenness -0.67504241

11 graph 1 NA Inattention_CAARS_Self_Zm Betweenness -0.32461056

12 graph 1 NA Hyperactivity_CAARS_Self_Zm Betweenness -0.25452419

13 graph 1 NA Selfconcept_CAARS_Self_Zm Betweenness -0.60495604

14 graph 1 NA Impulsivity_CAARS_Self_Zm Betweenness -0.04426508

15 graph 1 NA Infrequency_CAARS_Self_Zm Betweenness -0.60495604

16 graph 1 NA Inattention_CAARS_Obs_Zm Betweenness -0.60495604

17 graph 1 NA Hyperactivity_CAARS_Obs_Zm Betweenness 2.40875788

18 graph 1 NA Impulsivity_CAARS_Obs_Zm Betweenness 1.91815329

19 graph 1 NA Selfconcept_CAARS_Obs_Zm Betweenness -0.11435145

20 graph 1 NA TimeActQ Closeness -1.34506781

21 graph 1 NA DistQ Closeness -1.09204193

22 graph 1 NA AreaQ Closeness -1.24711840

23 graph 1 NA MicroEvQ Closeness -1.22818898

24 graph 1 NA SimplQ Closeness -1.39105984

25 graph 1 NA OmissQ Closeness NA

26 graph 1 NA CommQ Closeness NA

27 graph 1 NA RTQ Closeness NA

28 graph 1 NA RTVarQ Closeness NA

29 graph 1 NA ASTM_Zm Closeness NA

30 graph 1 NA Inattention_CAARS_Self_Zm Closeness 0.69326854

31 graph 1 NA Hyperactivity_CAARS_Self_Zm Closeness 0.85746756

32 graph 1 NA Selfconcept_CAARS_Self_Zm Closeness 0.32445309

33 graph 1 NA Impulsivity_CAARS_Self_Zm Closeness 0.62999835

34 graph 1 NA Infrequency_CAARS_Self_Zm Closeness 0.45099337

35 graph 1 NA Inattention_CAARS_Obs_Zm Closeness 0.68250038

36 graph 1 NA Hyperactivity_CAARS_Obs_Zm Closeness 1.10447216

37 graph 1 NA Impulsivity_CAARS_Obs_Zm Closeness 1.07599292

38 graph 1 NA Selfconcept_CAARS_Obs_Zm Closeness 0.48433057

39 graph 1 NA TimeActQ Strength -0.66421659

40 graph 1 NA DistQ Strength 0.15616159

41 graph 1 NA AreaQ Strength 0.24519955

42 graph 1 NA MicroEvQ Strength 0.81043011

43 graph 1 NA SimplQ Strength -0.63816656

44 graph 1 NA OmissQ Strength -1.48352671

45 graph 1 NA CommQ Strength -1.53719054

46 graph 1 NA RTQ Strength -0.69289939

47 graph 1 NA RTVarQ Strength 0.02976719

48 graph 1 NA ASTM_Zm Strength -2.25608852

49 graph 1 NA Inattention_CAARS_Self_Zm Strength 1.14169540

50 graph 1 NA Hyperactivity_CAARS_Self_Zm Strength 0.10756977

51 graph 1 NA Selfconcept_CAARS_Self_Zm Strength 0.36306961

52 graph 1 NA Impulsivity_CAARS_Self_Zm Strength 1.04830926

53 graph 1 NA Infrequency_CAARS_Self_Zm Strength 0.46240287

54 graph 1 NA Inattention_CAARS_Obs_Zm Strength 1.27429897

55 graph 1 NA Hyperactivity_CAARS_Obs_Zm Strength 0.23445609

56 graph 1 NA Impulsivity_CAARS_Obs_Zm Strength 1.35467340

57 graph 1 NA Selfconcept_CAARS_Obs_Zm Strength 0.04405450

58 graph 1 NA TimeActQ ExpectedInfluence -0.41732495

59 graph 1 NA DistQ ExpectedInfluence 0.33018253

60 graph 1 NA AreaQ ExpectedInfluence 1.00566039

61 graph 1 NA MicroEvQ ExpectedInfluence 1.43275888

62 graph 1 NA SimplQ ExpectedInfluence -1.09295498

63 graph 1 NA OmissQ ExpectedInfluence -1.35848173

64 graph 1 NA CommQ ExpectedInfluence -1.77744803

65 graph 1 NA RTQ ExpectedInfluence -0.97609310

66 graph 1 NA RTVarQ ExpectedInfluence 0.80118379

67 graph 1 NA ASTM_Zm ExpectedInfluence -2.09175511

68 graph 1 NA Inattention_CAARS_Self_Zm ExpectedInfluence 0.98840542

69 graph 1 NA Hyperactivity_CAARS_Self_Zm ExpectedInfluence 0.52159931

70 graph 1 NA Selfconcept_CAARS_Self_Zm ExpectedInfluence 0.33402250

71 graph 1 NA Impulsivity_CAARS_Self_Zm ExpectedInfluence 0.39187744

72 graph 1 NA Infrequency_CAARS_Self_Zm ExpectedInfluence 0.07266811

73 graph 1 NA Inattention_CAARS_Obs_Zm ExpectedInfluence 0.20426967

74 graph 1 NA Hyperactivity_CAARS_Obs_Zm ExpectedInfluence 0.51295332

75 graph 1 NA Impulsivity_CAARS_Obs_Zm ExpectedInfluence 0.68857970

76 graph 1 NA Selfconcept_CAARS_Obs_Zm ExpectedInfluence 0.42989684

Because of the separated network structure only strength and expected influence can be interpreted further.


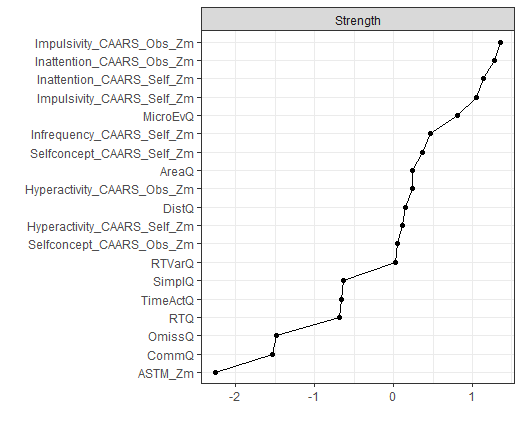


**Fig. S1. Node Strength**. Elevated standardized z-scores depicted on the x-axis signify increased strength, indicating that nodes with higher strength are more directly connected with other neuropsychological test variables in the network.

=== Correlation Stability Analysis ===

Sampling levels tested:

nPerson Drop% n

1 224 75.0 91

2 294 67.2 93

3 363 59.5 100

4 433 51.7 96

5 503 43.9 107

6 572 36.2 95

7 642 28.3 125

8 712 20.5 84

9 782 12.7 104

10 851 5.0 105

Maximum drop proportions to retain correlation of 0.7 in at least 95% of the samples:

expectedInfluence: 0.75 (CS-coefficient is highest level tested)

- For more accuracy, run bootnet(..., caseMin = 0.672, caseMax = 1)

strength: 0.75 (CS-coefficient is highest level tested)

- For more accuracy, run bootnet(..., caseMin = 0.672, caseMax = 1)

Accuracy can also be increased by increasing both 'nBoots' and 'caseN'.


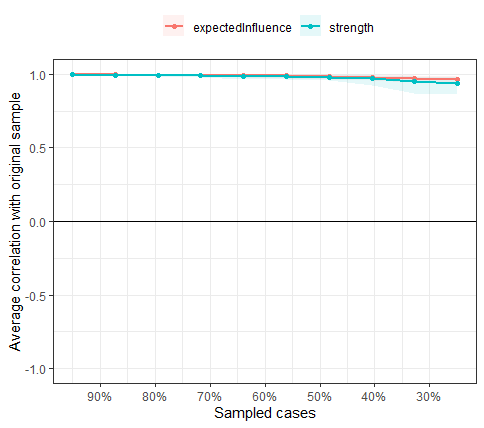


**Fig. S2.** Graphical display of correlation stability analyses of centrality measures expected influence and strength.


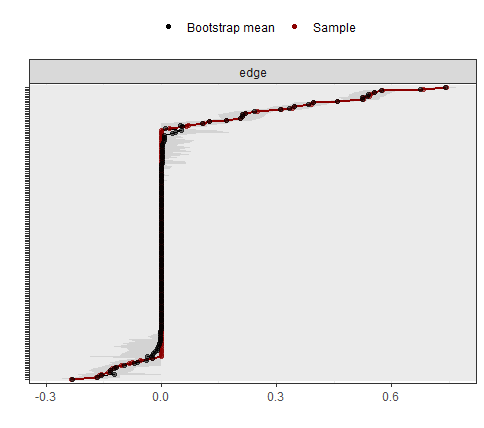


**Fig. S3.** Bootstrap confidence intervals (CIs) were generated for the estimated edge-weights within the network. Each horizontal line represents an individual edge in the network, arranged from the edge with the highest edge-weight to the one with the lowest edge-weight. The red line corresponds to the sample values of edge weights, while the black line represents the bootstrap mean values of edge weights. The shaded gray area indicates the bootstrapped CIs. For clarity, the y-axis labels have been omitted.


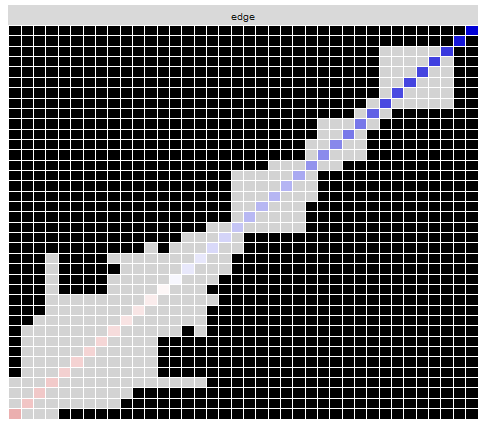


**Fig. S4.** Bootstrap difference tests (α=0.05) were conducted for edge boxes that exhibited non-zero values in the estimated network. In the results, gray boxes indicate nodes or edges that do not display a significant difference from each other, while black boxes represent nodes or edges with a significant difference. The color-coded boxes in the edge-weight plot align with the color of the corresponding edge in the network model.
